# Investigating genetic differentiation between brackish and fresh water collections of the arboviral vector *Aedes aegypti*

**DOI:** 10.1186/s13071-025-07239-3

**Published:** 2026-01-27

**Authors:** Dario Balcazar, Etowah Adams, Sinnathamby Noble Surendran, Ranjan Ramasamy, Jeffrey R. Powell, Andrea Gloria-Soria

**Affiliations:** 1 Department of Ecology and Evolutionary Biology, Yale University, New Haven, CT USA; 2 Department of Entomology, Center for Vector Biology & Zoonotic Diseases, The Connecticut Agricultural Experiment Station, New Haven, USA; 3 Centro de Estudios Parasitológicos y de Vectores (CEPAVE) CONICET—Universidad Nacional de La Plata, Boulevard 120 S/N Between Av. 60 and Calle 64, La Plata, Argentina; 4 Department of Molecular, Cellular, & Developmental Biology, Yale University, New Haven, CT USA; 5 Department of Zoology, University of Jaffna, Jaffna, Sri Lanka; 6 ID-FISH Technology, Milpitas, CA USA

**Keywords:** *Aedes aegypti*, Arboviral vector, Brackish water, Dengue, Genetics, Range expansion, Salinity tolerance

## Abstract

**Background:**

*Aedes aegypti* is typically regarded as a freshwater mosquito; however, recent studies have documented its development in brackish water habitats in coastal regions, including Sri Lanka’s Jaffna Peninsula. Compared with freshwater populations, brackish water samples in Jaffna display enhanced salt tolerance throughout larval-to-adult development, along with differences in gene expression, cuticle morphology and composition, and insecticide susceptibility.

**Methods:**

To explore the genetic basis of these differences, we performed a comparative genomic analysis using 5135 genome-wide single nucleotide polymorphisms (SNPs) from *Ae. aegypti* inhabiting freshwater and brackish water sites in the Jaffna Peninsula. Genetic diversity, population structure, and demographic parameters were evaluated using publicly available software. Candidate genomic regions potentially involved in salinity tolerance were identified through tests for environmental associations and genetic outlier detection.

**Results:**

After performing genotype quality control and first-degree relative removal on the initial 121 mosquitoes genotyped, the final dataset comprised 13 freshwater and 21 brackish water individuals. *Ae. aegypti* populations from the Jaffna Peninsula showed limited evidence of genetic structuring by collection site, with a subtle pattern associated with larval water salinity (distance-based redundancy analysis [dbRDA] *P* = 0.002, adjusted *R*^2^ = 0.01). Brackish-water populations displayed higher linkage disequilibrium, reduced effective population size, and lower nucleotide diversity relative to freshwater populations. Genetic outlier and environmental association analyses identified loci associated with fatty acid metabolism and other cellular pathways (e.g. Toll and Imd signaling pathways) as differentiated among the groups.

**Conclusions:**

We found subtle genetic differentiation between freshwater and brackish-water *Ae. aegypti* populations from the Jaffna Peninsula, suggesting that brackish-water populations may experience distinct evolutionary pressures potentially linked to adaptation to saline environments. Analyses point to fatty acid metabolism as one of the biological processes that could contribute to salinity tolerance in *Ae. aegypti*, possibly influencing cuticle modifications as a physiological response. Additional studies incorporating more collection sites and larger sample sizes for each salinity group are needed to further elucidate the mechanisms driving this differentiation. The ability of *Ae. aegypti* to adapt to brackish water substantially expands the range of potential larval sites it can occupy, particularly in coastal areas, and emphasizes the need to adjust vector control strategies accordingly.

**Graphical Abstract:**

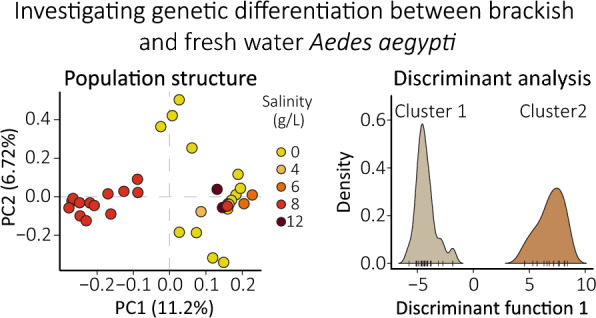

**Supplementary Information:**

The online version contains supplementary material available at 10.1186/s13071-025-07239-3.

## Background

*Aedes aegypti* is the principal vector of human arboviral diseases, including chikungunya, dengue, yellow fever, and Zika [1]. During its lifecycle, the species has been typically known to oviposit and undergo preimaginal development in freshwater (FW) habitats, ranging from water storage tanks to rock holes located near human settlements [1, 2]. Therefore, only FW habitats are presently targeted in arboviral disease control programs worldwide [2, 3].

**Fig. 1:**
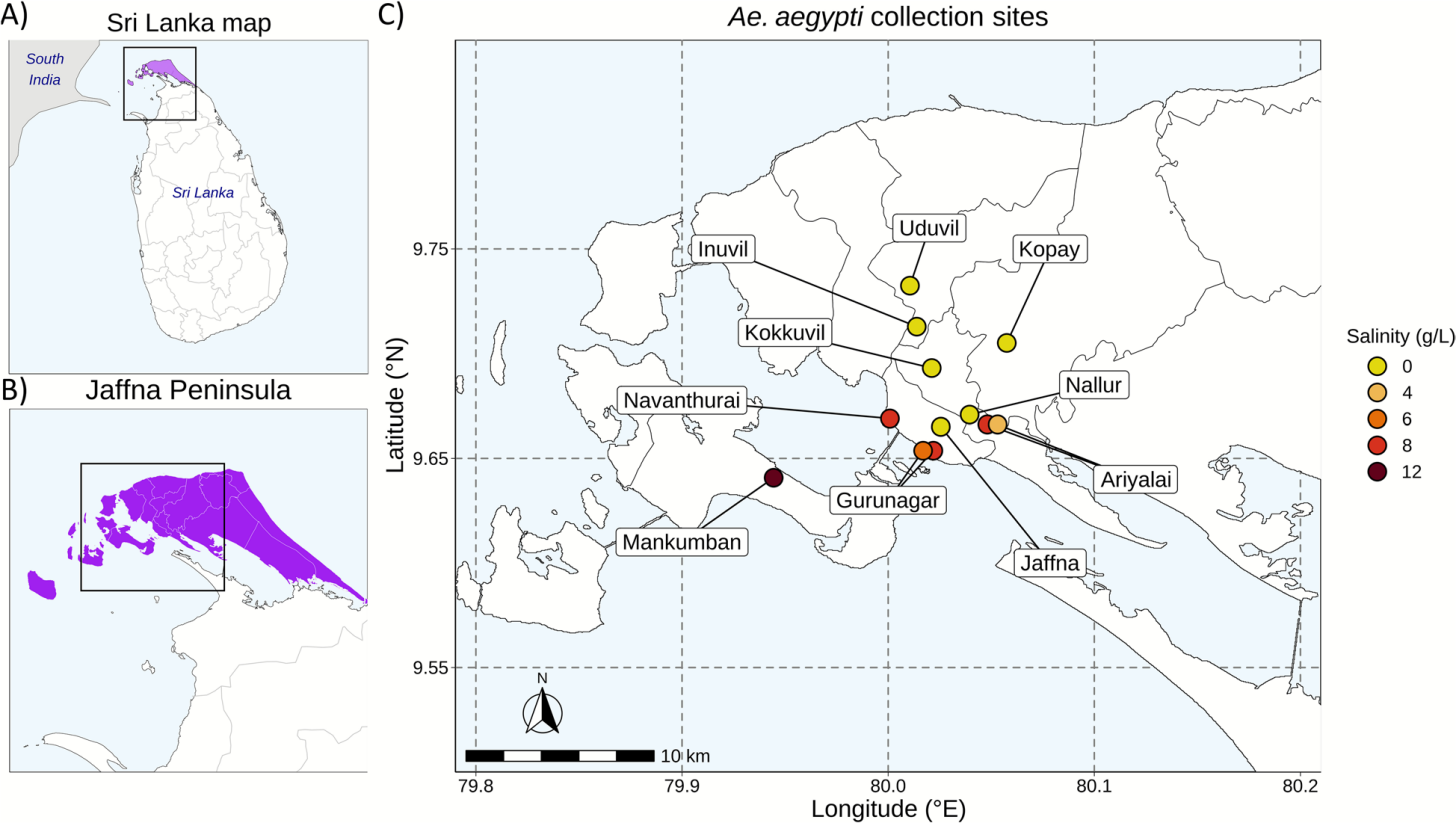
*Aedes aegypti* larval collection sites in Jaffna city and the Jaffna Peninsula, Sri Lanka. **A** Location of Sri Lanka in relation to South India. **B** The Jaffna Peninsula. **C** *Aedes aegypti* larval collection sites. Position and color of the dots highlight the collection sites and respective salinity level as follows: Ariyalai (8 and 4 g/L), Gurunagar (8 and 6 g/L), Navanthurai (8 g/L), and Mankumpan (12 g/L salt). Yellow circles represent freshwater (FW) sites (0 g/L salt), corresponding to Inuvil, Jaffna city center, Kokkuvil, Kopay, Nallur, and Uduvil

However, *Ae. aegypti* was recently shown to oviposit and undergo preimaginal development to adulthood in natural water collections with salinity up to 15 g/L in the 1100 km^2^ of the Jaffna Peninsula, Sri Lanka (Fig. 1) [4–10]. Salinity tolerant *Ae. aegypti* have also been observed in coastal Florida, USA [11]; Mexico [12]; and coastal areas of Brazil and Indonesia (details in reference [9]). Here, we define freshwater (FW), brackish water (BW), and saline water as containing < 0.5, 0.5–30, and > 30 g/L of salt, respectively [4]. The entire Jaffna Peninsula is considered a coastal zone, with an extensive coastline and multiple sea water inlets [13–15]. Given its small size (approximately 1100 km^2^), there is little variation in rainfall or temperature across the region. However, substantial variation in larval habitat salinity between sites across the peninsula, from inland to coastal areas, has been well documented [4, 5, 7, 14, 16]. The adaptation of *Ae. aegypti* to BW is of particular concern in the Jaffna Peninsula, as all sites are located at low elevation, within 10 km of the sea, and its groundwater aquifers are undergoing rapid salinization due to rising sea levels [13–15].

*Aedes aegypti* is the most important dengue vector in Jaffna city, with dengue incidence increasing during monsoonal rains [13, 17, 18]. BW *Ae. aegypti* can be infected with dengue virus and transmit it vertically (transovarially) to their progeny [19]. Virus-infected BW-dwelling *Ae. aegypti* may thus represent an unrecognized reservoir that contributes to dengue transmission at the onset of the monsoon season [19].

Substantial phenotypical differences are observed between BW and FW *Ae. aegypti* from the Jaffna Peninsula. BW mosquitoes exhibit higher salt tolerance, with a greater lethal dose concentration (LC50) for development from L1 larva to adult, compared with their FW counterparts [4, 5]. Moreover, FW *Ae. aegypti* displays a clear oviposition preference for FW over BW, while BW *Ae. aegypti* does not [5]. A recent multi-omics study comparing BW and FW *Ae. aegypti* revealed differential expression patterns in genes encoding cuticle-associated proteins, as well as distinct cuticle proteomic profiles in L4 larvae [6]. Thicker and modified procuticles, along with elevated levels of long chain hydrocarbons in the epicuticle, have been proposed to reduce water efflux and ion influx in larvae from BW mosquitoes [6, 8], and have also been associated with greater insecticide resistance [8, 9]. While these findings hinted at potential physiological mechanisms driving salinity tolerance, the precise genetic mechanisms responsible for this adaptation have remained elusive. In this work, we investigate the genetic differentiation between *Ae. aegypti* collected from BW and FW habitats in the Jaffna Peninsula using the *Ae. aegypti* single nucleotide polymorphism (SNP) chip [20]. By combining environmental association analyses and population genomic approaches, we provide evidence of genomic regions potentially involved in salinity tolerance in this species.

## Methods

### Mosquito collections

*Aedes* larvae were collected from Jaffna city and surrounding locations in the southwest of the Jaffna Peninsula and a nearby island (Fig. 1) from April to October 2020. Preimaginal stages were collected from a variety of habitats, including discarded plastic containers, open cement water storage tanks, etc. Conventional black plastic ovitraps were also used with water from nearby sources and placed in residential areas, as previously described [16]. The salinity of the water was measured with a hand-held refracto-salinometer (Atago Co. Ltd., Tokyo, Japan). Larvae were brought to the laboratory and reared to adulthood in their source water and identified at the species level with a standard taxonomic key [21]. Emergent adults identified as *Ae. aegypti* were tagged as either from FW (< 0.5 g/L salt) or BW (≥ 0.5 g/L salt) origin on the basis of the salinity of their larval site and preserved at −20 °C for further analysis. Details of the collection sites and water characteristics are provided in Electronic Supplemental Material (ESM) Table S1.

### DNA extraction, genotyping, and data quality filtering

DNA was extracted from individual adult mosquitoes, using the Qiagen DNeasy Blood and Tissue Kit (Qiagen) following the manufacturer’s instructions, with an additional RNAse A (Qiagen) step. Samples were stored at −20°C until further use.

Individuals were genotyped using the *Ae. aegypti* Axiom_aegypti1 SNP chip [20] (Life Technologies Corporation CAT#550,481) at the University of North Carolina Functional Genomics Core, Chapel Hill. Data files sent to Yale University were processed with the Affymetrix Axiom Analysis Suite 3.1 (Life Technologies–Thermo Fisher Scientific) to filter out samples with low quality (dish quality control [DQC] ≥ 0.82, quality control [QC] call rate ≥ 66, average call rate for passing samples ≥ 90) and to call the genotypes. PLINK v1.9 [22] was used over the SNP data to first remove SNPs that did not appear in > 80% of the individuals (missing rate per SNP [–geno 0.2]), followed by the exclusion of variants with a minor allele frequency below 0.01 [–maf 0.01]. Individuals with less than 5% missing SNPs were retained (missing rate per individual [–mind 0.05]).

### Kinship analysis and sibling removal

Individuals collected from the same water source could be related because female *Ae. aegypti* lay multiple eggs at a time. Inclusion of related individuals can bias certain demographic parameters [23]; thus, removal of close kin was performed. Sibling mosquitoes were excluded through pairwise comparisons using the KING kinship coefficient, calculated with VCFtools (v0.1.16, flag –relatedness2) [24]. A KING kinship coefficient threshold of 0.177 was used as the lower limit to identify close-related samples (first-degree relationship; full siblings). For each pair exceeding this threshold, one individual was removed.

We note here that, although removal of siblings is common and was performed in this study, it can pose certain challenges such as resulting in reduced sample size and making populations look infinitely large [25].

### Population structure analysis

The dataset after sibling removal contained 34 samples and was quality filtered a second time, as described above, with an additional step to remove variants in linkage disequilibrium (LD; –indep-pairwise 50 10 0.3). At the end, 5135 variants remained for subsequent analysis (ESM Fig. S1A).

To evaluate the contribution of salinity to the observed genetic structure, we conducted two complementary distance-based redundancy analyses (dbRDA) in R (vegan v2.7–2), including geographic coordinates as covariates to control for spatial structure [29]. Individual-based Euclidean genetic distances were computed using the *vegdist()* function [29], applied to a numeric genotype matrix (0, 1, 2) derived from genome-wide SNP data, and model significance was tested by 999 permutations, reporting adjusted *R*^2^ values. In a second dbRDA, spatial autocorrelation was explicitly modeled using Moran’s eigenvector maps (MEM; MEM1 and MEM2) derived from pairwise geographic distances (adespatial v0.3–2 R package) [30]. Stepwise model selection identified the predictors (salinity, MEM1, MEM2) contributing most to the explained genetic variation on the basis of adjusted *R*^2^ and permutation *P*-values.

Finally, we used Migrate-n (v5.0.6) [31, 32] software to test the possibility of gene flow between FW and BW samples. Using a Bayesian coalescent-based framework, Migrate-n is employed to estimate different demographic parameters. To reduce computational demands, we analyzed three independent datasets, each consisting of 500 randomly selected SNPs along our samples. Input files for Migrate-n were generated using the vcfR R package [33]. We compared four demographic models: (1) no gene flow, (2) unidirectional gene flow from FW to BW, (3) unidirectional gene flow from BW to FW, and (4) symmetric bidirectional gene flow. Each analysis assumed the Jukes and Cantor 1969 model (JC69) mutation model (transition/transversion ratio = 2.0) with a constant mutation rate and exponential priors for parameters (θ = 0.2, migration rate = 20, divergence time = 0.2). Posterior distributions were estimated using a Metropolis Markov chain Monte Carlo (MCMC) algorithm with a single long chain (2000 iterations, burn-in = 200, sampling increment = 500). Convergence was verified by effective sample sizes (ESS; > 1000) and low autocorrelation. Four heated chains (1, 1.5, 3.0, and 100,000) were used for Bezier-approximated thermodynamic integration to estimate log marginal likelihoods, with values closer to zero indicating better model fit.

### Comparative genomic analysis

Only samples collected at salinities of 0 g/L salt (FW) and 8 g/L salt (BW8) were included in subsequent comparative analyses aimed to identify candidate genetic regions related to salinity adaptation, as these groups had the largest sample sizes.

SNP genotypes were extracted from the previous dataset using PLINK 1.9. This dataset included 28 samples (13 and 15 mosquitoes for FW and BW, respectively) with 4112 variants (ESM Fig. S1A).

Using a dataset without LD pruning, a windowed genome-wide linkage disequilibrium (LD) was done in VCFtools (v0.1.16) [34] for each group, with flags –geno-r2 and –ld-window-bp 50,000. The squared correlation coefficient between SNPs (*r*^2^) was plotted against their distance in kilobases and fitted using a nonlinear regression model (nls) in R with the package ggpmisc (v0.6.1); regression line *R*^2^ [35]. To estimate the half-decay value for linkage disequilibrium distances (LD_50_)—defined as half of the maximum *r*^2^ in kb —and their 95% confidence intervals (CIs), we generated 100,000 predicted *r*^2^ values with each model. Bootstrapping (1000 replicates) was then applied by randomly sampling 0.2% of these values. Next, NeEstimator v2 [36] was used to infer the effective population size per group on the basis of the LD method, along with the 95% confidence intervals.

Group nucleotide diversity was calculated with the R package pegas (v1.3) [37], using bootstrapping to estimate 95% confidence intervals. *P*-values were calculated using a two-sided Wilcoxon test.

A genomic scan was conducted as pairwise *F*st comparisons between the FW and BW8 groups, using non-overlapping windows spanning 1 Mb in VCFtools (–weir-fst-pop, –fst-window-size 1,000,000, –fst-window-step 1,000,000). The visualization was created in R. Significant regions were selected using a threshold defined as the median plus two standard deviations (SD) (threshold = x̄ + 2 × SD), and only those containing at least three variants were considered.

VCFtools was used to estimate windowed nucleotide diversity (Pi) (–window-pi 1,000,000, –window-pi-step 1,000,000) and Tajima’s D (–TajimaD 1,000,000) along the chromosomes from each salinity group, with non-overlapping windows. Windows with at least three SNPs were considered. Linkage disequilibrium was then calculated from relevant genomic windows using the GWLD R package [38], on the basis of the *r*^2^ method.

Candidate SNPs for local adaptation were identified independently using PCAdapt (v4.3.5) [39]. This method does not require predefined population assignments. The software applies a PCA to our genetic data and partitions the total genetic variation into *K* principal components (PCs). It then evaluates the correlation between SNPs and the retained PCs, detecting differentially distributed variants across the samples. Mahalanobis distance was used to calculate *P*-values, and a conservative approach was applied by using the auto-calibrated *P*-values. To control the false positives, *P*-values were transformed into *q*-values, and the false discovery rate (FDR) threshold was set to 0.01.

Finally, a genomic–environmental association test between the genomic data and salinity measured in the collection sites was conducted using LFMM2 (v1.1) (Fig. 1) [40] . In our case, auto-calibrated *P*-values were not used, since a strong over-correction was detected. Uncalibrated *P*-values were extracted and manually calibrated by using a genomic inflation factor (GIF) of 0.8. While this value may be less stringent, it increases the sensitivity of the analysis to detect potentially relevant variants. False positives were controlled by converting *P*-values into *q*-values, and the FDR threshold was set to 0.0001. Only those variants, genes, and Kyoto Encyclopedia of Genes and Genomes (KEGG) pathways identified through LFMM2 that were also supported by *F*st scan or PCAdapt are described here.

The number of genetic clusters for PCAdapt and LFMM2 analyses was set to *K* = 2 on the basis of the admixture and DAPC results.

### Identification of candidate genes

SNPs identified as significant across all genomic scans were annotated using BCFtools (v1.16) [41] and SnpEff (v5.2) [42], respectively. The *Ae. aegypti* LVP AGWG genome was used as a reference for SnpEff, [43].

Gene information including coordinates, annotations and gene ontology (GO) terms were obtained from National Center for Biotechnology Information (NCBI)-Gene [44], VectorBase databases [45], and the Gene Ontology repository [46, 47]. KEGG pathway data [48] were accessed through the R package KEGGREST (v1.46.0) [49]. The metabolic map (code: aag01100) of *Ae. aegypti* retrieved from the KEGG database was plotted using the package ggkegg (v1.4.1) [50].

SNP coordinates were verified using the updated SNP chip coordinates provided in [51]. Coordinates were transformed into a BED file and cross-referenced with the *Ae. aegypti* GFF file from VectorBase using BEDTools (v2.30.0) [52] to verify the annotation performed with SnpEff. Descriptions for each gene associated with a significant SNP were manually retrieved from Entrez from NCBI-Gene and KEGG databases. Only genes with complete functional annotations were retained for representation and were cross-referenced with the RNA-seq analysis from Ramasamy et al. (2021) [6] to assess expression levels in *Ae. aegypti* populations from BW and FW environments in Sri Lanka. Two expression categories were defined on the basis of the fold-change (FC) values reported by the authors: high expression (FC ≥ 10) and moderately high expression (FC ≥ 4 and < 10) in BW L4 compared with FW L4.

Allele frequencies were compared between the groups with a two-sided chi-squared test using the prop.test function in base R [53].

## Results

### Population structure among *Ae. aegypti* BW and FW collections in Sri Lanka

After applying data quality filtering, 106 of the original 121 samples were retained. Subsequent removal of first-degree relationships (siblings) yielded 34 mosquitoes (FW = 13; BW = 21) (ESM Fig. S1A, B; ESM Table S1), harboring 5135 variants for further analysis.

**Fig. 2:**
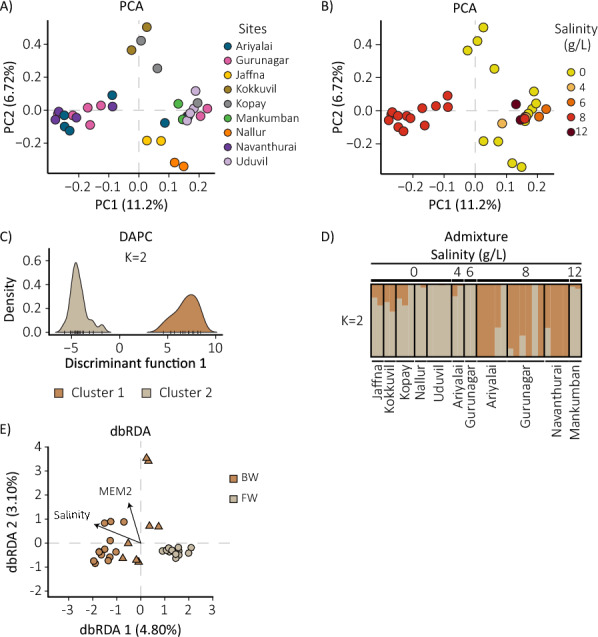
Genetic structure of *Aedes aegypti* from Jaffna based on collection sites and their salinity. **A** and **B** Principal component analysis including 34 first-degree filtered *Ae. aegypti* mosquitoes, with colors representing **A**) collection sites and **B**) salinity levels. Each sample is represented by a dot. The proportion of total variation explained by each principal component is indicated. **C** Genetic clusters identified by discriminant analysis of principal components (DAPC). Density axis represents the proportion of total mosquitoes in each genetic cluster, while discriminant function 1 axis refers to the first and only discriminant function. Cluster 1 (brown shadow) includes BW individuals collected at salinities of 8 g/L, whereas cluster 2 (light brown shadow) includes FW samples, and mosquitoes from Ariyalai (4 g/L and one from 8 g/L), Gurunagar (6 g/L and one from 8 g/L), and Mankumban (12 g/L). **D** Admixture analysis at *K* = 2 clusters. The height of the color bar represents the proportion of each sample assigned to one of the identified genetic clusters. Individuals with ancestry from multiple genetic clusters are represented by bars with varying color proportions. Black horizontal bars above the plot delineate the boundaries between different salinity (g/L) conditions. **E** Distance-based redundancy analysis (dbRDA) showing the relationship between salinity and genetic structure, with spatial effects modeled by Moran’s eigenvector maps. MEM2 was retained as the second-best predictor, although it was not statistically significant (*P* > 0.05). The first two dbRDA axes explained 44.2% and 28.2% of the constrained variance, corresponding to 4.8% and 3.1% of the total genetic variance, respectively. Salinity was the only significant predictor of genetic differentiation (*P* = 0.002). Arrow length indicates the direction and relative correlation of each predictor with the ordination axes but does not represent the absolute strength of its contribution to the model

Principal component analysis (PCA) on the complete dataset did not cluster samples by collection site (Fig. 2A; ESM Fig. S2). However, some clustering was observed by salinity, particularly of those mosquitoes collected at sites with 8 g/L salt (Fig. 2B). All FW samples were included in a broader cluster, though mosquitoes from different salinity levels were also present there, including samples from Ariyalai (4 g/L and one from 8 g/L salt), Gurunagar (6 g/L and one from 8 g/L salt), and two samples from Mankumban (12 g/L salt).

Using DAPC, we identified two as the optimal number of genetic clusters present in our data (ESM Fig. S3A). One cluster (Fig. 2C, brown shadow) primarily comprised individuals from BW (8 g/L), while the second cluster ( Fig. 2C, light-brown shadow) included FW samples along with the BW individuals identified in the PCA (Fig. 2C). Cross-validation of error in the admixture analysis to determine the optimal number of genetic groups was not conclusive, unable to differentiate between the presence of *K* = 1 or 2 optimal genetic clusters (ESM Fig. S3B). However, at *K* = 2 the groups recapitulated the two groupings recovered in the DAPC and the PCA (Fig. 2D).

To evaluate the relationship between salinity and population genetic structure, we performed a distance-based redundancy analysis (dbRDA), including latitude and longitude as covariates to account for geographic structure. After controlling for spatial covariation, salinity explained 3.9% of the total genetic variance (adjusted *R*^2^ = 0.01; *F* = 1.32; *P* = 0.001). In a second dbRDA, which included salinity and the first two Moran’s eigenvector maps (MEM1 and MEM2) as predictors, the model explained 10.9% of the total genetic variance (adjusted *R*^2^ = 0.02; *F* = 1.22; *P* = 0.002). Stepwise model selection retained salinity as the only significant predictor (*F* = 1.48; *P* = 0.002), indicating that salinity was the main environmental factor associated with genetic differentiation, whereas neither MEM1 nor MEM2 were significant (*P* > 0.05).

Finally, different migration models were tested to explain the presence of FW genotypes in samples collected from BW populations. As shown in ESM Fig. S4, the model indicating bidirectional gene flow between the two groups provided the best fit, showing the highest log marginal likelihood (log(ml) = –9731.19).

### Genetic diversity of *Ae. aegypti* from FW (0 g/L salt) and BW8 (8 g/L salt)

To explore the underlying genetic differentiation between salinity conditions, we focused on the two groups with sufficient sample sizes: freshwater (FW; 0 g/L) and brackish water (BW8, 8 g/L). Individuals collected at 8 g/L but clustering with FW genotypes were retained in the BW8 group to capture all phenotypic variation within the groups.

**Fig. 3:**
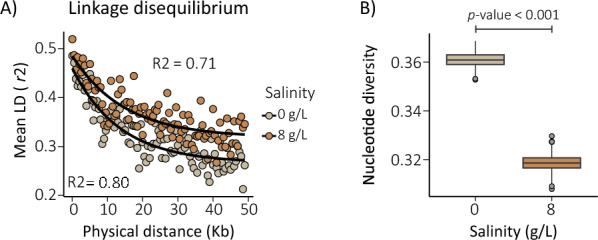
Genetic differentiation between FW (0 g/L) and BW8 (8 g/L) mosquitoes from the Jaffna Peninsula. **A** Mean pairwise linkage disequilibrium (*r*^2^) in samples collected from freshwater (FW; 0 g/L) and brackish water (BW8, 8 g/L). **B** Genome-wide nucleotide diversity for each group. *P*-values were calculated using a two-sided Wilcoxon test. Brown highlights BW8 individuals (collected at 8 g/L of salt), whereas light brown only indicates FW samples

Linkage disequilibrium (LD) analysis revealed higher LD values in BW8 compared with FW (LD₅₀_FW_: 9.54 kb, CI: 9.04–9.75 kb; LD₅₀_BW8_: 10.50 kb, CI: 9.91–10.71 kb) (Fig. 3A; ESM Fig. S3B, left panel; ESM Table S2). Consistent with this pattern, effective population size was lower in BW8 (*Ne* = 12.2, 95% CI: 12.1–12.2; ESM Fig. S5A) compared with FW (*Ne* = 31, 95% CI: 30.9–31.1; ESM Fig. S5A). Overall nucleotide diversity was significantly higher in FW samples than in BW8 (nucleotide diversity: FW 0.36, 95% CI: 0.33–0.39 and BW8 0.31, 95% CI: 0.29–0.33; Wilcoxon rank sum test, *W* = 0; *P* < 0.0001; Fig. 3B).

### Selection scans

**Fig. 4:**
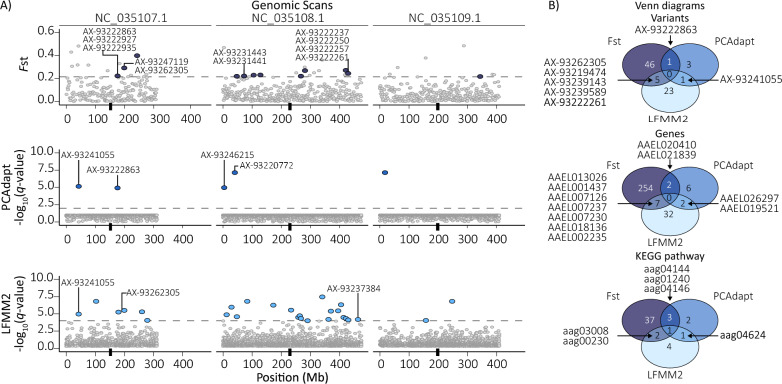
Genomic scans and functional analysis of freshwater and brackish water (8 g/L) *Aedes aegypti*. **A** Results from the pairwise *F*st comparison (top), PCAdapt (middle), and LFMM2 (bottom) scans. Colored dots above the threshold (dashed line, *F*st = 0.21) indicate significant 1 Mb regions (dark blue dots for the *F*st scan) or significant individual variants (PCAdapt and LFMM2, with blue and light blue dots above thresholds of 2 and 3, respectively). The black boxes along the position axis mark the positions of the centromeres. **B** Venn diagram showing common variants (top subpanel), genes (middle subpanel), and pathways (bottom subpanel) associated with SNPs detected by all three methods

*F*st pairwise comparisons detected 12 genetically differentiated regions between the FW and BW8 groups (Fig. 4A, upper panel), including 52 unique variants, most of which were annotated as intergenic (24.7%) or upstream of genes (20.6%) (ESM Fig. S6A). Analysis with PCAdapt identified five explanatory variants, while salinity-association analysis using LFMM2 detected 29 SNPs associated to salinity tolerance (Fig. 4A, middle and bottom panels, respectively). In both cases, intronic (PCAdapt = 42.9%, LFMM2 = 38.5%) and intergenic (PCAdapt = 42.9%, LFMM2 = 17.9%) variants were predominant (ESM Fig. S6B, C; ESM Tables S3–S5).

Within the 12 regions identified as outliers in the *F*st scan, there were 263 annotated genes, while the variants identified with PCAdapt and LFMM2 were associated with 10 and 41 genes, respectively (Tables S6–S8). Neither of the variants nor genes were detected by the three methods, but seven variants and 11 genes were identified by at least two of three scans (Fig. 4B, Variants and Genes panels). The GO term distributions are shown in ESM Figs. S7 and S8. Although the *ppk301* gene (AAEL000582), which encodes a sodium channel protein, has been identified as a key regulator of *Ae. aegypti* egg-laying preference for FW over BW habitats [54], it did not exhibit differentiation between our two genetic clusters, nor were associated allele frequencies different (FW = 0.40 versus BW8 = 0.28; *χ*^2^ = 3.18, *df* = 1, *P* = 0.074).

Due to the lack of gene convergence among the three methods, we used the KEGG pathway database to obtain evidence of the candidate genes’ potential functionality. This analysis revealed six pathways involved in at least two of the genomic scans (Fig. 4B, KEGG pathway panel; ESM Fig. S9), which are depicted in ESM Fig. S10A: ribosome biogenesis in eukaryotes, Toll and Imd signaling pathways, peroxisome, and endocytosis. Genes retrieved by all three methods were involved in metabolism (ESM Fig. S10A, KEGG pathway panel), with the metabolic pathway encompassing 21 distinct cellular metabolic processes. ESM Fig. S10B presents the *Ae. aegypti* metabolic map, highlighting the pathways in which the identified genes are involved. Most of these pathways were detected by the *F*st scan, with purine metabolism also identified by LFMM2 and thiamine/folate metabolism by PCAdapt.

We cross-referenced the identified KEGG-associated genes with differentially expressed genes identified previously between *Ae. aegypti* mosquitoes from BW versus FW habitats in Sri Lanka [6]. Most of the cellular pathways identified in our analyses contained genes whose transcription was upregulated in BW mosquitoes (ESM Fig. S10A), with two genes exhibiting particularly high expression levels: AAEL026297 (SNP ID: AX-93241055), associated with the Toll and Imd signaling pathway, and AAEL004913 (SNP IDs: AX-93231443 and AX-93231441), associated with endocytosis.

**Fig. 5:**
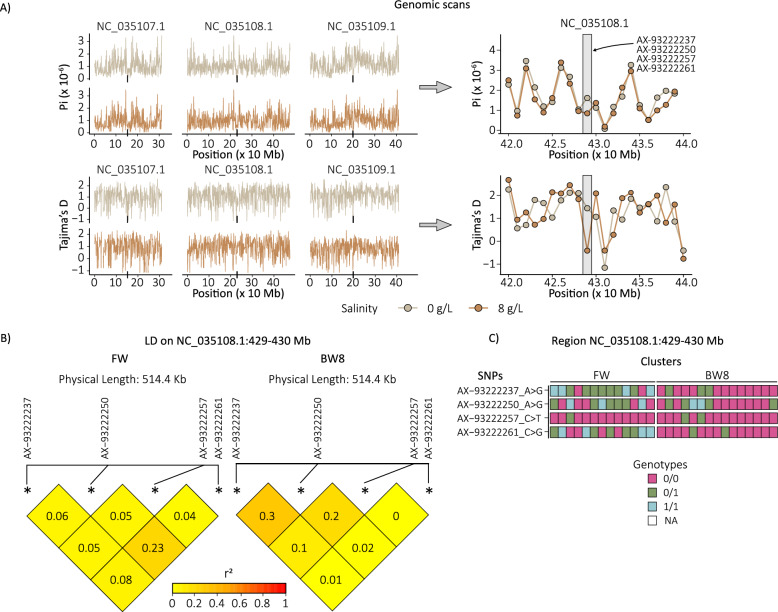
Genomic regions associated with local adaptation between mosquitoes from freshwater and brackish (8 g/L) environments. **A** Nucleotide diversity (Pi, top panel) and Tajima’s *D* (bottom panel) calculated for FW (light-brown lines and dots) and BW8 (brown lines and dots) groups in 1 Mb non-overlapping windows. The left-hand plots show all chromosomes, while the right-hand plots focus on region NC_035108.1:410–450 Mb. The black boxes along the position axis in the left panel mark the centromeres. In the right panel, the dots indicate the central position of each genomic window, while the grey box highlights the window NC_035108.1:429–430 Mb, showing the identity of the sites within this region. **B** Linkage disequilibrium in the genomic region NC_035108.1:429–430 Mb. **C** SNP genotypes in the genomic region NC_035108.1:429–430 Mb for individual samples in each cluster. Dark pink boxes indicate reference homozygotes, green boxes indicate heterozygotes, and light blue boxes indicate alternative homozygotes

Although our scans identified two genomic regions that contained two or more genes associated with a cellular pathway—NC_035107.1:170–180 Mb (AAEL021839 and AAEL002419) and NC_035108.1:429–430 Mb (AAEL002228 and AAEL002237)—differences in Pi and Tajima’s *D* between FW and BW8 mosquitoes were detected only within the latter (Fig. 5A, grey box in the right panel). Within this genomic region, both nucleotide diversity (FW = 1.6 × 10^−6^ versus BW8 = 8.5 × 10^−7^) and Tajima’s *D* (FW = 1.44 versus BW8 = −0.41) were lower in BW8 mosquitoes. In this context, Fig. 5C shows an increased frequency of the reference alleles (AX-93222237_A > G, AX-93222250_A > G, AX-93222257_C > T, AX-93222261_C > G) in BW8 samples compared with FW samples (observed frequency of reference alleles: FW = 0.615, BW8 = 0.875; *χ*^2^ = 18.89, *df* = 1, *P* < 0.0001). Moreover, linkage disequilibrium is higher in this genomic region in BW8 samples (Fig. 5B), with a mean *R*^2^ of 0.11 compared with 0.085 in FW.

## Discussion

Adaptation of mosquito vectors to novel habitats is an emerging ecological trend that facilitates their geographic expansion and may significantly impact transmission dynamics of vector-borne diseases [55]. One recent and relevant example is the rapid expansion of *Anopheles stephensi*, a typical FW malaria vector, from India to northern Sri Lanka in 2017 and from the Middle East to countries in the Horn of Africa in 2012–2022 [56]. Emerging evidence shows that *An. stephensi* can develop in BW, potentially facilitating its spread into new coastal and urban habitats in Sri Lanka [56, 57]. Recent reports also show that *Ae. aegypti* can develop in brackish water, suggesting a similar potential for expansion [4–12]. In this study, we present preliminary evidence suggesting that *Ae. aegypti* populations inhabiting BW environments may exhibit genetic differences relative to FW populations, possibly reflecting local responses to salinity variation across the Jaffna Peninsula. [15].

### Brackish-water environments may influence the genetic structure of *Ae. aegypti* in Jaffna

Analysis of population structure did not reveal clustering of samples based on collection sites. However, we found evidence of some genetic clustering associated with habitat salinity. In particular, mosquitoes derived from BW at a salt concentration of 8 g/L (Ariyalai, Gurunagar, and Navanthurai) formed a well-defined cluster independent of location. A broader cluster encompassed mosquitoes collected from multiple FW habitats and additional BW habitats (Fig. 2). Genetic clustering of FW genotypes with a proportion of some mosquitoes collected in BW could be attributed to migration between the environments, facilitated by the short distance between collection sites (mean ± SD: 5.4 ± 3.5 km), despite the preference of FW-derived *Ae. aegypti* for ovipositing in FW [5]. This scenario is further supported by several lines of evidence: (1) the absence of a definitive reproductive or oviposition barrier between FW and BW populations [5]; (2) support for a model including bidirectional gene flow; (3) tolerance of FW samples to salinity up to 8 g/L, with survival gradually declining to total mortality at 16 g/L [5]; (4) the *ppk301* gene showing no significant differences in allele frequency between groups in our study; and (5) the observation of no differences in *ppk301* expression levels between the two conditions [6].

Taken together, and despite ongoing gene flow, FW and BW populations show signs of incipient genetic differentiation, with salinity likely playing a role in shaping their genetic structure. These observed patterns may reflect demographic processes such as a bottleneck, evidenced by increased linkage disequilibrium, reduced effective population size, and lower nucleotide diversity (Fig. 3), and support the hypothesis that BW-adapted individuals have repeatedly originated from FW populations. Understanding how these demographic and adaptive processes interact will be essential to better anticipate *Ae. aegypti*’s responses to changing environmental conditions.

### Putative genomic signals of selection under brackish-water conditions: Insights into fatty acid metabolism

The insect cuticle serves as a critical barrier against water and ion loss in aquatic preimaginal stages and prevents desiccation in adult *Ae. aegypti* [58] and other insects [59, 60]. This protective function is largely attributed to the epicuticle, the outermost layer of the cuticle, which is rich in very long-chain hydrocarbons and waxy esters. Thickening of the procuticle and increased epicuticular hydrocarbon content are thought to contribute significantly to the adaptation of mosquito larvae to BW environments. The biosynthesis of these hydrocarbons involves fatty acid synthesis by a multi-enzyme fatty acid synthase complex, elongation, and subsequent modification by a NADH/NADPH-dependent fatty acid acyl CoA reductase and insect-specific cytochrome P450 enzymes of the 4G1 family (described first in Drosophila [61]). Expression levels of a fatty acid synthase (AAEL002228), a very long-chain fatty acid elongase (AAEL024147), a fatty acyl CoA reductase (AAEL008125), and two potential cytochrome P450 decarbonylases (AAEL004054 and AAEL006824), are markedly upregulated in *Ae. aegypti* L4 larvae reared in BW compared with FW conditions [6, 9].

Using genomic scans, we found evidence for an additional peroxisomal fatty acyl-CoA reductase gene (AAEL001737) showing genetic differentiation between FW and BW8 and previously reported as moderately upregulated in BW samples [6]. Our study also suggests that the NC_035108.1:429–430 Mb genomic region could be subject to selection pressure in BW8 samples. This genomic region appears to be relevant to fatty acid metabolism pathways, as it encodes three (AAEL002228, AAEL002237, and AAEL022506) of the five fatty acid synthase genes that have been described in *Ae. aegypti* [62] and has been previously reported to be upregulated in response to salinity stress [6]. Thicker cuticle and protein composition changes in BW *Ae. aeygpti* has been previously described [6, 8, 9]. Selection signals related to fatty acid metabolism, especially within this genomic region, suggest that adaptive changes supporting larval survival in BW environments might involve a genetically mediated, cuticle-based response to salinity. Consistent with the important role of the cuticle in mosquito adaptation to saline environments, a duplication of cuticle protein genes and an intronic deletion in a V-type proton pump gene were identified as potential mechanisms underlying adaptation to high salinity in the mosquito *An. stephensi* [63].

Cuticle modifications that reduce water loss are a general response to desiccating conditions such heat and drought. Because salinity can also induce osmotic stress, similar physiological pathways may be activated under different stressors. Major environmental differences other than salinity are unlikely to exist among the sites, given their proximity and the geographic extent of the Jaffna Peninsula, although other factors cannot be entirely ruled out.

### Limitations of the study and future implications for understanding *Ae. aegypti* salinity adaptation

This study was complicated by the high genetic relatedness of individuals collected from the same larval sites, which considerably reduced the sample size. The initial dataset comprised 121 *Ae. aegypti* larvae collected from coastal locations spanning both FW and BW habitats in the Jaffna Peninsula. From this initial number of mosquitoes, 87 samples (close to two thirds of our collection) were excluded due to a high degree of relatedness, likely resulting from single-female oviposition events within containers. The remaining 34 mosquitoes included 13 from FW habitats and 21 from natural BW habitats, spread among the different salinity groups. The limited number of mosquitoes available for genetic analysis, combined with the small size of the peninsula that allows *Ae. aegypti* to develop and oviposit across both habitat types, may have constrained the resolution and statistical power to detect subtle patterns of differentiation. Other limitations include: (1) the small number of samples representing the different salinity groups (4, 6, and 12 g/L; *n* = 2); (2) the assumption that individuals collected from BW larval sites were tolerant to BW, although it is possible that the salinity tolerance of BW-collected individuals clustering with FW genotypes was lower than that of BW genotypes, as described by Ramasamy et al. (2014) [5]; and (3) the low density of SNPs across the large *Ae. aegypti* genome, with most loci located in noncoding regions. Together, these limitations likely reduced the power of our analyses and constrained our ability to detect clear signals of salinity adaptation. Future studies with larger, independent samples, direct measures of salinity tolerance, and higher-resolution genomic data (e.g., next-generation sequencing [NGS]) will be essential to better understand *Ae. aegypti* adaptation to BW environments.

Although our study highlights pathways showing differential gene expression [6] and genetic differentiation as potential factors involved in BW adaptation, their specific roles remain to be confirmed. Fatty acid metabolism, in particular, appears as a promising candidate, given the upregulation of fatty acid synthases and the associated cuticle thickening observed in larvae and adult females from BW habitat [6–9]. These cuticle modifications are particularly relevant for vector control strategies, as they have been associated with increased resistance to larvicides and adulticides, respectively [8, 9].

Such efforts will improve our understanding of *Ae. aegypti* population structure in relation to salinity and inform more effective vector control, particularly as coastal brackish-water habitats become increasingly important in the species’ ecology and disease transmission [64].

## Conclusions

Our study on *Ae. aegypti* populations inhabiting FW and BW environments in the Jaffna Peninsula from Sri Lanka reveals a certain degree of genetic differentiation among groups, likely shaped by variations in salinity levels. Genomic analyses suggest signals of a demographic bottleneck and reduced genetic diversity in BW populations, possibly indicating demographic processes associated with adaptation to saline habitats. Notably, the enrichment of genes involved in fatty acid metabolism suggest that this pathway may play a role in facilitating survival in BW environments, correlating with previous findings of cuticle thickening as a key adaptive response, which in turn contributes to increased insecticide resistance. Understanding how this adaptative process takes place is key to prevent further *Ae. aegypti* range expansion. These findings emphasize the need to consider a wider range of aquatic habitats, particularly BW sites, in vector surveillance and control strategies, as they may host genetically distinct mosquito populations with varying levels of insecticide resistance, potentially shaping the dynamics of arboviral disease transmission and control.

## Supplementary Information

Supplementary file 1: Fig. S1. Sample filtering and kinship analysis of *Ae. aegypti* from Sri Lanka. (A) Schematic representation of the processing and filtering of *Ae. aegypti* mosquitoes from Sri Lanka, highlighting the number of samples retained at each step. (B) Kinship coefficient values for each pairwise comparison of Sri Lanka samples, categorized into different relationship levels on the basis of the authors’ recommendations: twins [0.354, 0.5), first-degree relatives [0.177, 0.354), second-degree relatives [0.0884, 0.177), third-degree relatives [0.0422, 0.0884), and unrelated individuals [−0.5, 0.0422). The pink circle indicates highly related samples that were removed from our datasets.

Supplementary file 2: Fig. S2. Collection sites of *Aedes aegypti* across the Jaffna Peninsula, Sri Lanka. Map showing the locations of *Aedes aegypti* collection sites across the Jaffna Peninsula. Circles represent the sampling sites, color-coded by locality name.

Supplementary file 3: Fig. S3. Selection of the optimal number of genetic clusters in *Ae. aegypti* from Sri Lanka. Different numbers of clusters (up to ten) were tested for our SNP data using both software. (A) In Adegenet, the optimal number of clusters was determined by comparing Bayesian information criterion (BIC) values across tests. (B) In admixture, the best number of clusters was selected on the basis of the cross-validation error across the tests. In both cases, the optimal number of clusters was determined by selecting the one with the lowest BIC or CV error.

Supplementary file 4: Fig. S4. Inference of gene flow between freshwater and brackish-water *Ae. aegypti. *Comparison of alternative migration models tested using Migrate-n. The four models represent (from left to right): (1) no gene flow; (2) unidirectional gene flow from FW to BW; (3) unidirectional gene flow from BW to FW; and (4) symmetric bidirectional gene flow. Model comparison was based on the Bezier-approximated log marginal likelihood, where values closer to zero indicate better model fit.

Supplementary file 5: Fig. S5. Effective population size and linkage disequilibrium decay across genetic clusters. (A) Effective population size for each genetic cluster. (B) LD_50_ distance (kb), calculated as half of *r*^2^_*max*_, derived from the data estimated in Fig. 2.

Supplementary file 6: Fig. S6. Analysis of SNPs identified with different genomic scans. After genomic scanning, SNP annotations were analyzed to determine the most represented types of variation: (A) *F*st scanning (window size 1 Mb), (B) PCAdapt, and /C) LFMM2 association tests.

Supplementary file 7: Fig. S7. Analysis of genes identified with *F*st genomic scans. Genes identified through *F*st genomic scans (window size 1 Mb) were analyzed to determine the different types of GO terms according to their ontology (biological process, cellular component, and molecular function, respectively)

Supplementary file 8: Fig. S8. Analysis of genes identified with PCAdapt and LFMM2 scans. Genes identified through (A) PCAdapt and (B) LFMM2 genomic scans (window size 1 Mb) were analyzed to determine the different types of GO terms according to their ontology (biological process, cellular component, and molecular function, respectively)

Supplementary file 9: Fig. S9. Biological pathways associated with genomic outliers across three detection methods. Genes detected through genomic scans with (A) *F*st, (B) PCAdapt, and (C) LFMM2 were analyzed using the KEGG pathway database to identify the pathways they are involved in and to determine the most represented.

Supplementary file 10: Fig. S10. Exploring KEGG pathway relationships among candidate genes under putative selection. (A) Network diagram illustrating the connections between KEGG pathways (gray circles) and the genes identified by the *F*<sub>ST</sub> scan (dark blue circles), PCAdapt (blue circles), and LFMM2 (light blue circles). Color-partitioned circles indicate genes detected by more than one approach. Solid black lines represent confirmed connections between genes and pathways based on GFF annotations and the KEGG database, whereas dashed lines denote indirect or putative associations inferred from gene function. (B) *Ae. aegypti* metabolic map retrieved from the KEGG database. Colored dots and lines represent metabolites and their biochemical connections, respectively. Annotations correspond to the names of each metabolic route. Green arrows indicate changes in gene expression levels (fold change [FC]) from RNA-seq data in [6], where solid green arrows denote high expression (FC ≥ 10) and lighter green arrows denote moderate expression (4 ≤ FC < 10).

Supplementary file 11: Table S1. *Aedes aegypti* larval collection sites and habitat characteristics. The initial number of samples corresponds to the number of processed mosquitoes for each collection site, while the final number of samples indicates the number of mosquitoes retained after genotype quality control and first-degree samples removal. Table S2. Linkage disequilibrium measures in *Ae. aegypti* populations from FW (0 g/L) and BW8 (8 g/L) habitats. Table S3. SNPs identified as candidates for selection on the basis of *F*st outlier analysis. Table S4. SNPs identified as candidates for selection on the basis of PCAdapt outlier analysis. Table S5. SNPs identified as candidates for selection on the basis of LFMM2 outlier analysis, using salt concentration as environmental variable. Table S6. Gene annotations for positively selected genomic regions detected by *F*st scans. Table S7. Gene annotations for positively selected genomic regions detected by PCAdapt scan. Table S8. Gene annotations for positively selected genomic regions detected by LFMM2 scan using salt concentration as environmental variable.

Supplementary file 12.

## Data Availability

Data are provided in the supplementary information files. The VCF file contains the inferred genotypes obtained from the SNP-chip platform. This dataset was pre-filtered to retain sites missing in less than 20% of individuals and with a minor allele frequency above 0.01. Moreover, only individuals with less than 5% missing SNPs were retained.

## Abbreviations

BW: Brackish water
BW8: Brackish water at 8 g/L of salt
FW: Freshwater
PCA: Principal component analysis
DAPC: Discriminant analysis of principal components
dbRDA: Distance-based redundancy analysis
MEM: Moran’s eigenvector maps
SNP: Single nucleotide polymorphism
LD_50_: Half-decay value for linkage disequilibrium distances
r^2^: Squared correlation coefficient between SNPs
