# Supplementary figures and images for: Investigating genetic differentiation between brackish and fresh water collections of the arboviral vector Aedes aegypti
Source: Parasit Vectors. 2026 Jan 27;19:91. doi: 10.1186/s13071-025-07239-3 (PMC12915009; doi:10.1186/s13071-025-07239-3)

A)

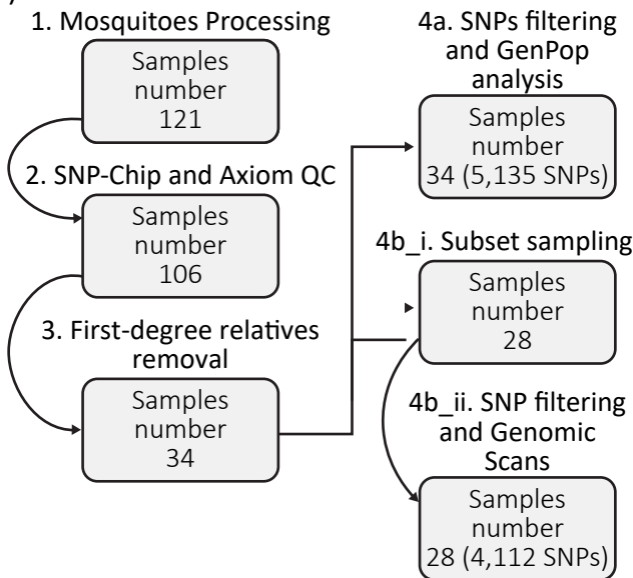

B)

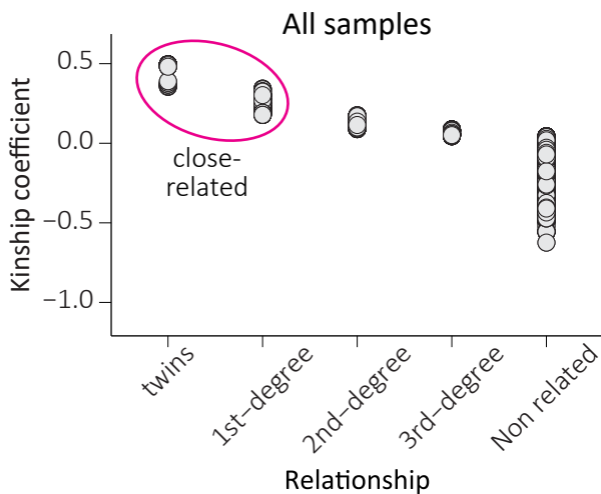

Supplement: Supplementary file 1 — Fig. S1. Sample filtering and kinship analysis of Ae. aegypti from Sri Lanka. (A) Schematic representation of the processing and filtering of Ae. aegypti mosquitoes from Sri Lanka, highlighting the number of samples retained at each step. (B) Kinship coefficient values for each pairwise comparison of Sri Lanka samples, categorized into different relationship levels on the basis of the authors’ recommendations: twins [0.354, 0.5), first-degree relatives [0.177, 0.354), second-degree relatives [0.0884, 0.177), third-degree relatives [0.0422, 0.0884), and unrelated individuals [−0.5, 0.0422). The pink circle indicates highly related samples that were removed from our datasets. [file 13071_2025_7239_MOESM1_ESM.pdf]

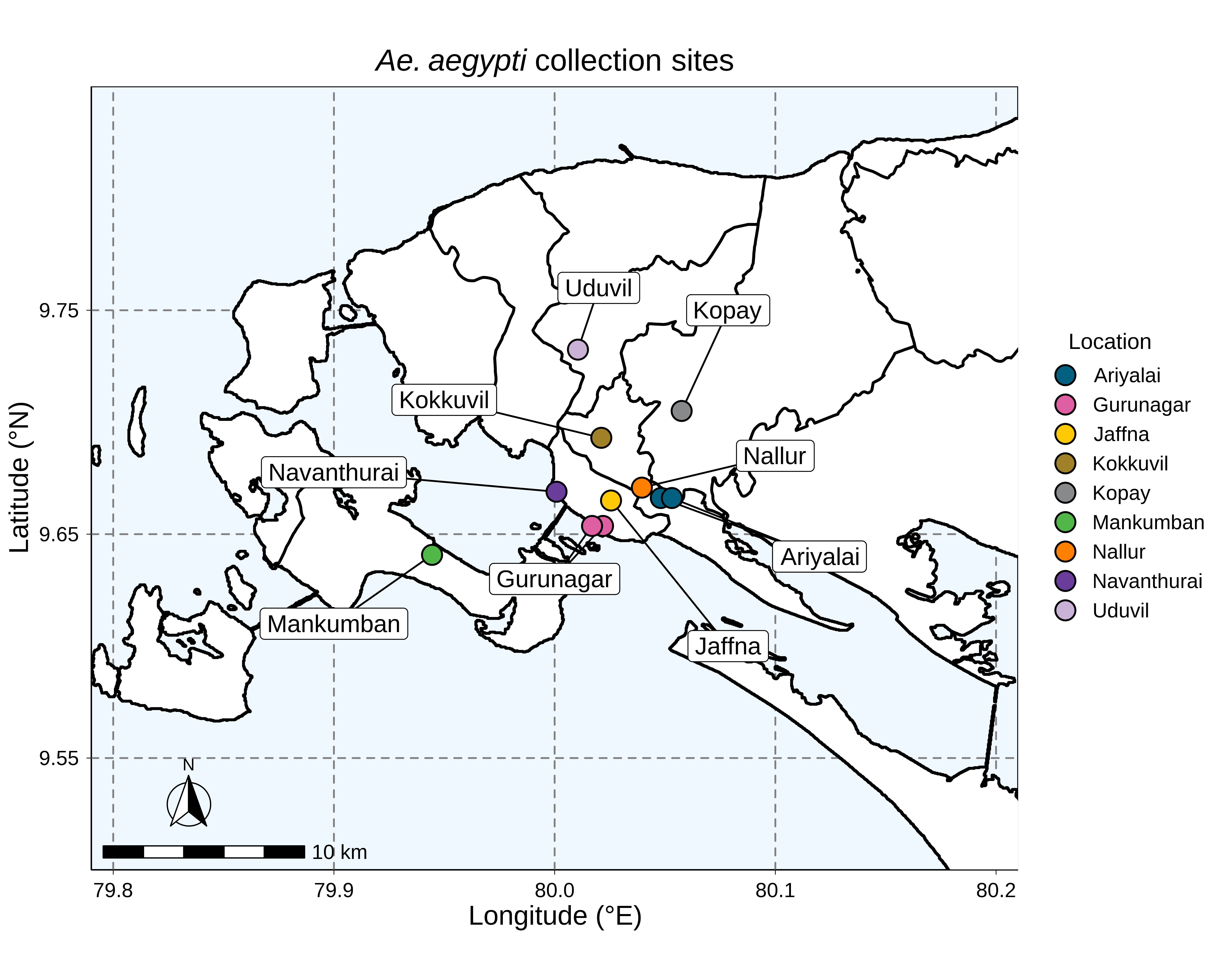

Supplement: Supplementary file 2 — Fig. S2. Collection sites of Aedes aegypti across the Jaffna Peninsula, Sri Lanka. Map showing the locations of Aedes aegypti collection sites across the Jaffna Peninsula. Circles represent the sampling sites, color-coded by locality name. [file 13071_2025_7239_MOESM2_ESM.png]

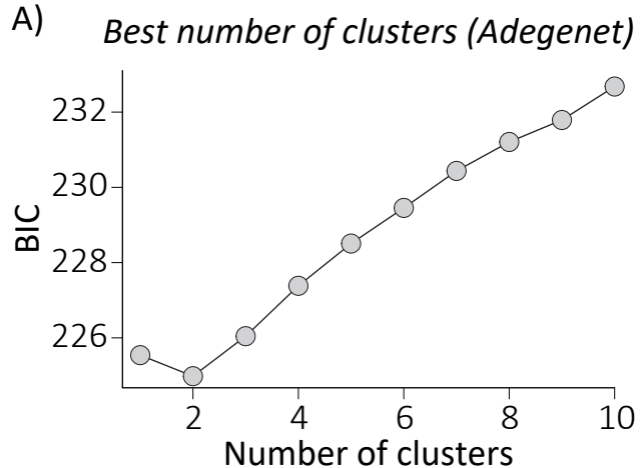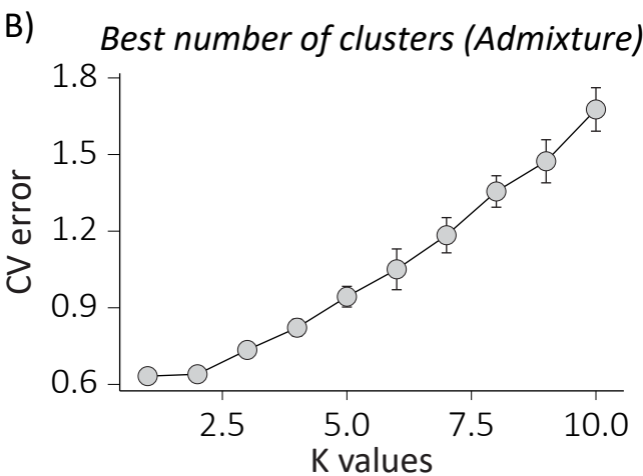

Supplement: Supplementary file 3 — Fig. S3. Selection of the optimal number of genetic clusters in Ae. aegypti from Sri Lanka. Different numbers of clusters (up to ten) were tested for our SNP data using both software. (A) In Adegenet, the optimal number of clusters was determined by comparing Bayesian information criterion (BIC) values across tests. (B) In admixture, the best number of clusters was selected on the basis of the cross-validation error across the tests. In both cases, the optimal number of clusters was determined by selecting the one with the lowest BIC or CV error. [file 13071_2025_7239_MOESM3_ESM.pdf]

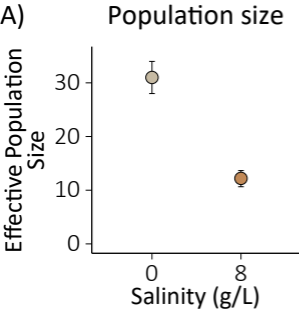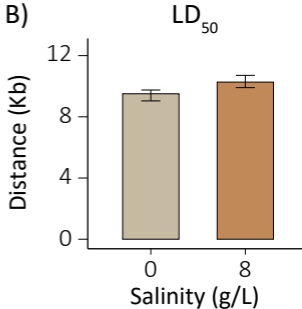

Supplement: Supplementary file 5 — Fig. S5. Effective population size and linkage disequilibrium decay across genetic clusters. (A) Effective population size for each genetic cluster. (B) LD50 distance (kb), calculated as half of r2max, derived from the data estimated in Fig. 2. [file 13071_2025_7239_MOESM5_ESM.pdf]

# SNPs annotation

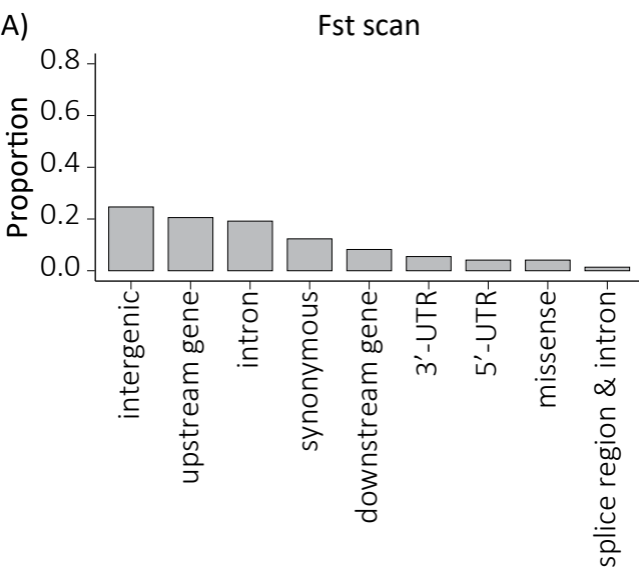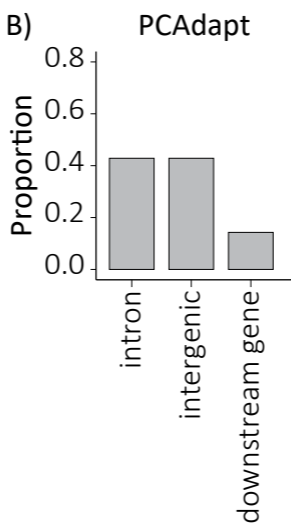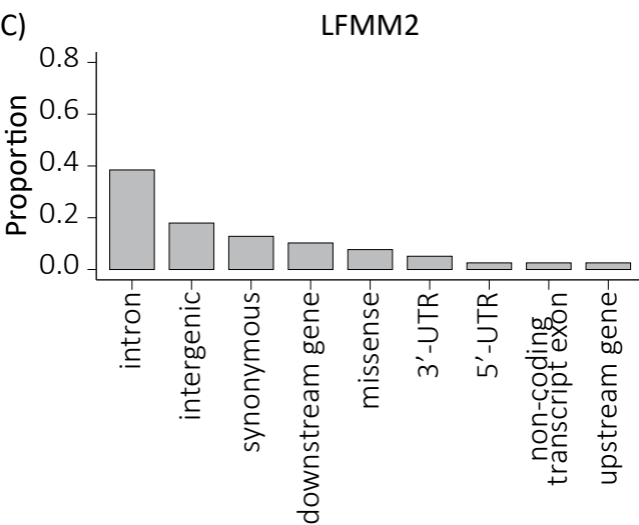

Supplement: Supplementary file 6 — Fig. S6. Analysis of SNPs identified with different genomic scans. After genomic scanning, SNP annotations were analyzed to determine the most represented types of variation: (A) Fst scanning (window size 1 Mb), (B) PCAdapt, and /C) LFMM2 association tests. [file 13071_2025_7239_MOESM6_ESM.pdf]

# GO analysis (Fst scan)

## Biological Process

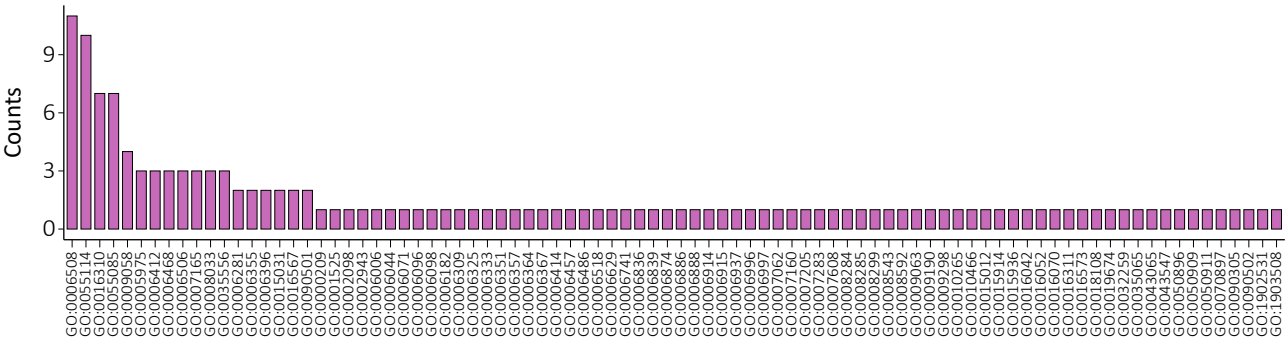

## Cellular Component

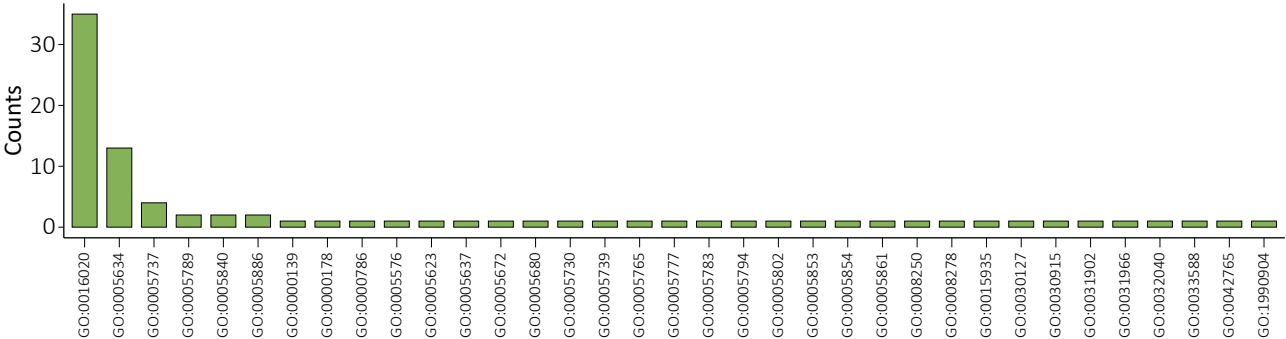

## Molecular Function

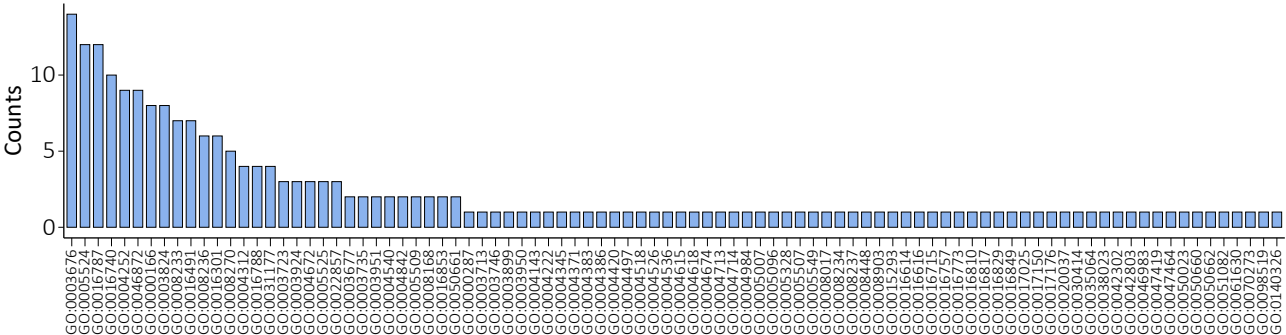

Supplement: Supplementary file 7 — Fig. S7. Analysis of genes identified with Fst genomic scans. Genes identified through Fst genomic scans (window size 1 Mb) were analyzed to determine the different types of GO terms according to their ontology (biological process, cellular component, and molecular function, respectively) [file 13071_2025_7239_MOESM7_ESM.pdf]

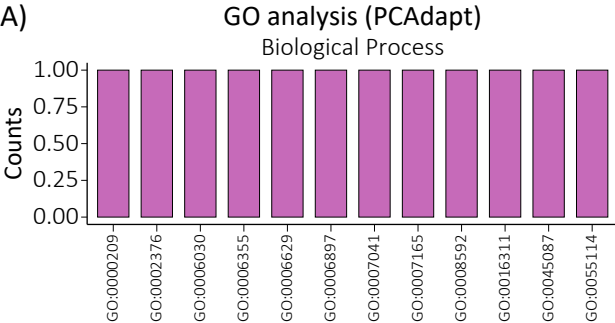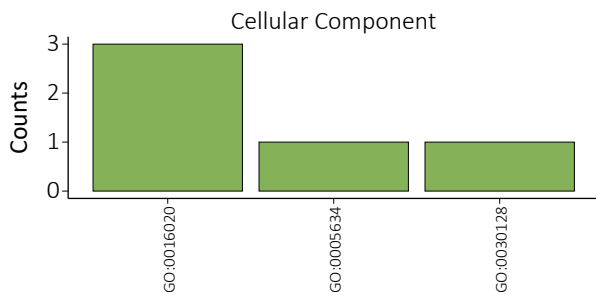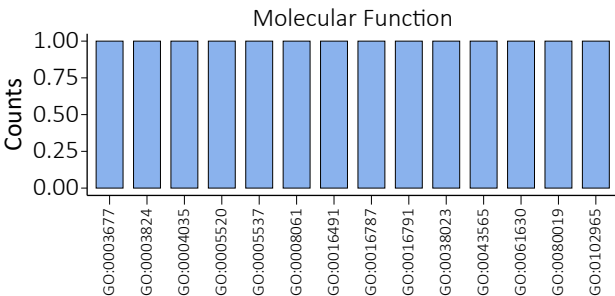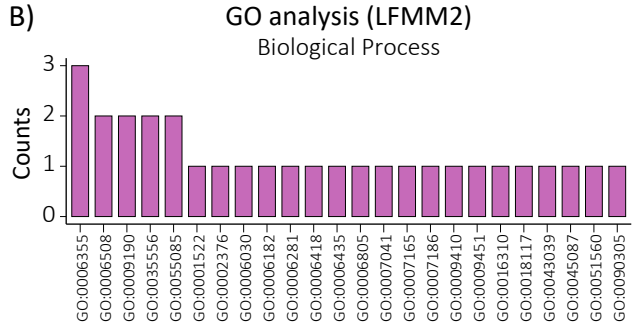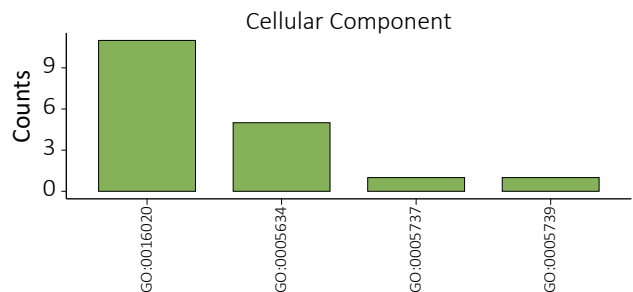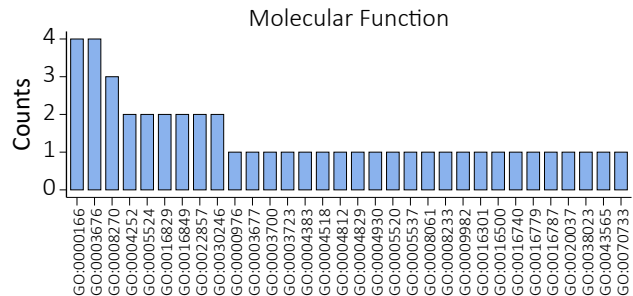

Supplement: Supplementary file 8 — Fig. S8. Analysis of genes identified with PCAdapt and LFMM2 scans. Genes identified through (A) PCAdapt and (B) LFMM2 genomic scans (window size 1 Mb) were analyzed to determine the different types of GO terms according to their ontology (biological process, cellular component, and molecular function, respectively) [file 13071_2025_7239_MOESM8_ESM.pdf]

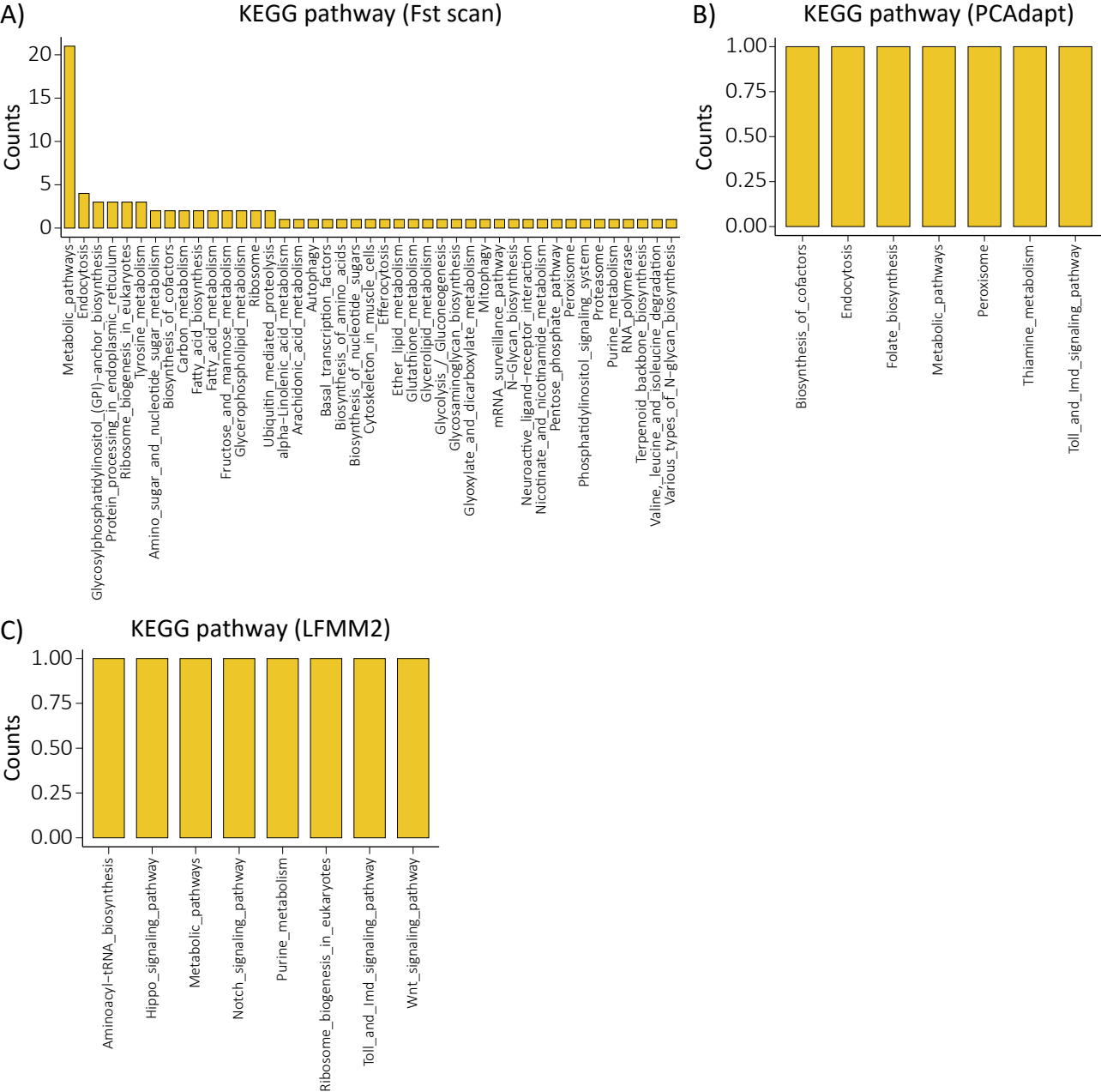

Supplement: Supplementary file 9 — Fig. S9. Biological pathways associated with genomic outliers across three detection methods. Genes detected through genomic scans with (A) Fst, (B) PCAdapt, and (C) LFMM2 were analyzed using the KEGG pathway database to identify the pathways they are involved in and to determine the most represented. [file 13071_2025_7239_MOESM9_ESM.pdf]

A)

## KEGG pathways

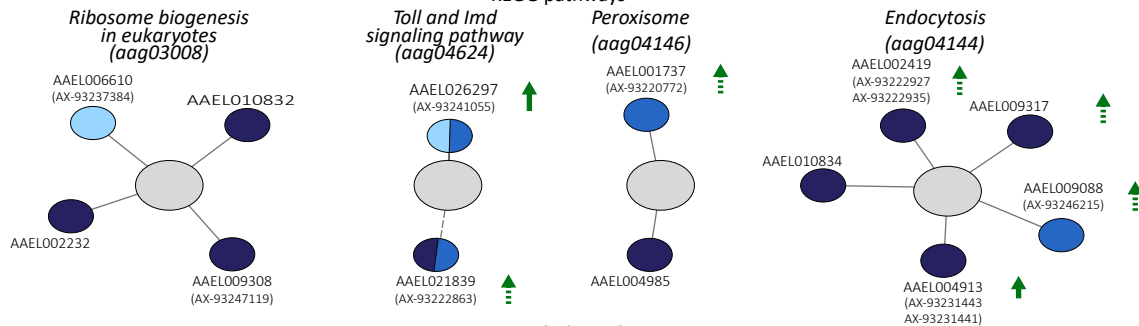

B)

## KEGG Metabolic pathways

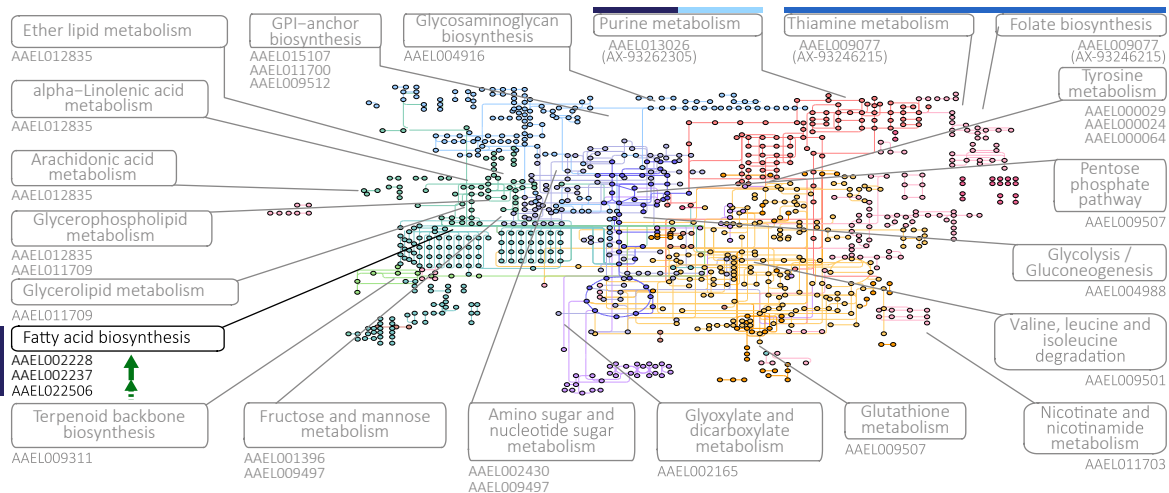

Fold-change estimated in Ramasamy et al., 2021

▲ FC >= 10   ▲ 4 <= FC < 10

Supplement: Supplementary file 10 — Fig. S10. Exploring KEGG pathway relationships among candidate genes under putative selection. (A) Network diagram illustrating the connections between KEGG pathways (gray circles) and the genes identified by the FST scan (dark blue circles), PCAdapt (blue circles), and LFMM2 (light blue circles). Color-partitioned circles indicate genes detected by more than one approach. Solid black lines represent confirmed connections between genes and pathways based on GFF annotations and the KEGG database, whereas dashed lines denote indirect or putative associations inferred from gene function. (B) Ae. aegypti metabolic map retrieved from the KEGG database. Colored dots and lines represent metabolites and their biochemical connections, respectively. Annotations correspond to the names of each metabolic route. Green arrows indicate changes in gene expression levels (fold change [FC]) from RNA-seq data in [6], where solid green arrows denote high expression (FC ≥ 10) and lighter green arrows denote moderate expression (4 ≤ FC < 10). [file 13071_2025_7239_MOESM10_ESM.pdf]
