# Supplementary material for: Investigating genetic differentiation between brackish and fresh water collections of the arboviral vector Aedes aegypti
Source: Parasit Vectors. 2026 Jan 27;19:91. doi: 10.1186/s13071-025-07239-3 (PMC12915009; doi:10.1186/s13071-025-07239-3)
Supplement: Supplementary file 4 — Fig. S4. Inference of gene flow between freshwater and brackish-water Ae. aegypti. Comparison of alternative migration models tested using Migrate-n. The four models represent (from left to right): (1) no gene flow; (2) unidirectional gene flow from FW to BW; (3) unidirectional gene flow from BW to FW; and (4) symmetric bidirectional gene flow. Model comparison was based on the Bezier-approximated log marginal likelihood, where values closer to zero indicate better model fit. [file 13071_2025_7239_MOESM4_ESM.pdf]

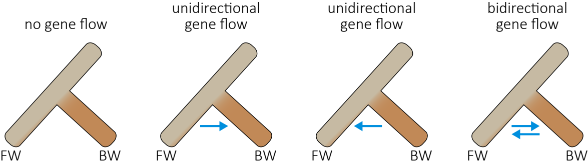

log(marginal likelihood)			
-10,350.08	-10,081.08	-9,981.39	-9,731.19
